# Gene Expression Analysis of Alfalfa Seedlings Response to Acid-Aluminum

**DOI:** 10.1155/2016/2095195

**Published:** 2016-12-15

**Authors:** Peng Zhou, Liantai Su, Aimin Lv, Shengyin Wang, Bingru Huang, Yuan An

**Affiliations:** ^1^School of Agriculture and Biology, Shanghai Jiao Tong University, Shanghai 200240, China; ^2^Department of Plant Biology and Pathology, Rutgers University, New Brunswick, NJ 08901, USA; ^3^Key Laboratory of Urban Agriculture (South), Ministry of Agriculture, Shanghai 201101, China

## Abstract

Acid-Aluminum (Al) is toxic to plants and greatly affects crop production worldwide. To understand the responses of plants to acid soils and Aluminum toxicity, we examined global gene expression using microarray data in alfalfa seedlings with the treatment of acid-Aluminum. 3,926 genes that were identified significantly up- or downregulated in response to Al^3+^ ions with pH 4.5 treatment, 66.33% of which were found in roots. Their functional categories were mainly involved with phytohormone regulation, reactive oxygen species, and transporters. Both gene ontology (GO) enrichment and KEGG analysis indicated that phenylpropanoid biosynthesis, phenylalanine metabolism, and flavonoid biosynthesis played a critical role on defense to Aluminum stress in alfalfa. In addition, we found that transcription factors such as the MYB and WRKY family proteins may be also involved in the regulation of reactive oxygen species reactions and flavonoid biosynthesis. Thus, the finding of global gene expression profile provided insights into the mechanisms of plant defense to acid-Al stress in alfalfa. Understanding the key regulatory genes and pathways would be advantageous for improving crop production not only in alfalfa but also in other crops under acid-Aluminum stress.

## 1. Introduction

Aluminum (Al) combined with acid is the main factor limiting plant growth and crop production worldwide [1]. Al in soils is solubilized into ionic forms, especially when the soil pH falls to lower than 5. Roots are the primary targets of acid-Al toxicity in plants. Several studies have reported Al inhibition of cell elongation and cell division in plant roots [2–4]. The root apex (particularly the distal transition zone of the root) has been shown to be a critical site for the perception of Al toxicity [5]. Zhou et al. [6] reported the presence of Al ions in cell walls, intracellular membranes, and the center of the nucleus in alfalfa root cells. Furthermore, extensive research has demonstrated that Al^3+^ alters physiological processes (i.e., cytosolic Ca^2+^ homeostasis and cytoskeleton dynamics) and modifies the levels of endogenous nitric oxide in the root tips [7–9].

Al-induced toxicity is caused by the high binding affinity of Al to various extracellular and intracellular substances. Most reports have suggested that organic acids (OAs) play an important role in the mechanism by which plants tolerate Al stress [10]. Plants also have other mechanisms to cope with Al stress. Phenolic compounds such as flavonoids, alkaloids, terpenoids, and glycosides form strong complexes with Al ions, and these compounds have been implicated in internal Al detoxification in* Camellia sinensis* and other Al-accumulating species [11, 12]. Kidd et al. [13] reported that differential Al-tolerance in* Zea mays* genotypes showed a better correlation with the rate of Al-stimulated root exudation of flavonoids (catechin and quercetin) than with Al-activated exudation of OAs. Other studies showed that the induction of antiperoxidation enzymes could ameliorate the oxidative damage caused by Al stress and lead to Al-tolerance phenotypes in various plants [14, 15].

Many genes and signaling pathways have been proposed to be involved in the Al stress response in plants [16–19]. A group of Al-induced genes, such as* wali1–5* in wheat* (Triticum aestivum)*,* Sali5-4a* and* Sali3-2* in soybeans* (Glycine max)*, and* ALS3* in* Arabidopsis*, have been identified and characterized [20–22].* Medicago sativa* L. (alfalfa) is very sensitive to acid and Al ions. The alfalfa yield in acidic soils was inhibited due to reduced nitrogen fixation and destroying symbiotic bacteria [23]. However, the underlying mechanism of Aluminum phytotoxicity on root growth at the molecular level remains unclear. Here, we used microarray analysis to investigate genome-wide transcriptional profiling and bioinformatics data mining to examine the enriched gene ontology and metabolic pathways. The identified genes, which is differentially expressed under Al stress, together with the metabolic pathway information obtained from microarray analysis, will provide an informative platform for cultivating Al-tolerant species with improved agronomic features in the future.

## 2. Materials and Methods

### 2.1. Plant Material and Treatment

Alfalfa (WL-525), which is an Al-tolerant cultivar [24, 25], was obtained from the National Seed Corporation (New Delhi, India). Healthy seeds of uniform size were surface-sterilized with 0.5% (v/v) sodium hypochlorite solution and repeatedly washed with double-distilled water. After drying with a blotting paper, the seeds were placed on two layers of filter paper in a petri dish. The filter paper was soaked in 2 mL of 0.2 mM CaCl_2_ solution containing 0 (pH 6.0), 0 (pH 4.5), 0.8 (pH 4.5), or 3.2 (pH 4.5) mM AlCl_3_. The pH was adjusted by the addition of 1 M HCl. The experiments were conducted in an environmentally controlled growth room with 14 h/27°C day and 10 h/25°C night cycles, light intensity of 480 *μ*mol·m^2^·s^−1^, and relative humidity of 70 ± 5%. After germination for 60 h, the seedlings with green cotyledons and formed roots were defined as being successfully germinated and survived. Seedlings without green cotyledons or formed roots were considered dead and failed germination. We then calculated the germination and survival rate according to these definitions. All the experiments were repeated three times. The whole seedlings (with roots, stems and leaves) germinated in the presence of 0 (pH 6.0), 0 (pH 4.5), 0.8 (pH 4.5), and 3.2 (pH 4.5) mM of AlCl_3_ were collected, frozen in liquid nitrogen for 7 min, and then stored at −80°C for microarray analysis.

### 2.2. Microarray Analysis

Seedlings germinated in the presence of 0 (pH 6.0), 0 (pH 4.5), 0.8 (pH 4.5), or 3.2 (pH 4.5) mM AlCl_3_ for 60 h were collected and used for microarray analysis. Total RNA was extracted using the TRIzol Reagent (Invitrogen, Carlsbad, CA, USA) from the germinated alfalfa samples. The quality and integrity of the total RNA were evaluated with an Agilent 2100 Bioanalyzer (Agilent Technologies, Inc. Santa Clara, USA) (with OD_260 nm_/OD_280 nm_ ≥ 1.8 and RIN ≥ 9.0). RNA purification and microarray hybridization were performed according to the one-color microarray-based gene expression analysis protocol. RNA samples (3 *μ*g) extracted from each individual were pooled to form four sets (referred to as seedlings germinated under 0 (pH 6.0), 0 (pH 4.5), 0.8 (pH 4.5), or 3.2 (pH 4.5) mM AlCl_3_ solution for 60 h). The RNA pool was used as a template for cDNA preparation. cDNA was further transcribed into cRNA and double-labeled using an Agilent low RNA input fluorescent linear amplification kit (Agilent Technologies, Santa Clara, CA, USA). Then, 0.5 *μ*g of labeled cRNA samples was purified, mixed with hybridization buffer, and hybridized to oligonucleotide microarrays (Medicago Gene Expression 4 × 44K; Agilent, Santa Clara, CA, USA; http://www.genomics.agilent.com/) for 17 hours at 65°C. The microarrays were designed based on RefSeq (Release 32), UniGene (Build 33), TIGR Plant Transcript Assemblies (Release 2), and TIGR Gene Indices (Release 9) and contained a total of 43,803 oligonucleotide probes (60-mer). After hybridization, the slide glass was washed using a gene expression wash buffer kit (Agilent) and scanned with the Genepix 400B (Axon Instruments, Foster City, CA, USA). The fluorescence intensity was calculated using Feature Extraction software version 9.5 (Agilent), and the data were analyzed with GeneSpring GX software version 11.0 (Agilent). The whole experiments were biologically repeated three times, and the microarray data were normalized by GeneSpring GX 11.0.

Transcripts with more than twofold differences between the seedlings grown under Al stress at a specified statistical cutoff (fold change [FC] ≥ 2.0 and *P* < 0.05 according to the *t* test) were defined as differentially expressed genes.

### 2.3. Quantitative Real-Time RT-PCR (qPCR)

To validate our microarray results, total RNA was extracted from the alfalfa seedlings germinated with different concentrations of AlCl_3_ solution using the TRIzol Reagent (Invitrogen, Carlsbad, CA, USA). First-strand cDNA was generated with a RETROscript kit (Invitrogen) using oligo (dT) primers and the RETROscript RTase. Then, we examined 17 genes that were differentially up- or downregulated following Al^3+^ treatments (FC ≥ 2.0 and *P* < 0.05* t* test) using qPCR. 17 genes which were mainly involved in the phenylpropanoid and flavonoid biosynthesis pathways (based on AgriGO and KEGG analysis) or transcription factors (TFs) that might be related to metabolic pathways [26, 27]. The specific primers used for qPCR were listed in Table 1. These primers were used to validate differentially expressed genes (FC ≥ 2.0 and *P* < 0.05;* t* test) obtained from microarray analysis. Briefly, the 25 *μ*L qPCR amplification mixture contained 25 ng of template cDNA, 12.5 *μ*L of 2x SYBR Green I Master Mix buffer (Applied Biosystems), and 300 nM each of the forward and reverse primers. The reactions were run on an ABI Prism 5700 sequence detector (Applied Biosystems). The PCR protocol was as follows: polymerase activation and predenaturation for 4 min at 94°C, followed by 40 cycles at 94°C for 30 s, 58°C for 30 s, and 72°C for 30 s. We selected three genes (EF-*α*, 18S rRNA, and ubiquitin; primers listed in Table 1, accession numbers: XM_003618727 in Genbank, DQ311983 in Genbank, and TC174254 in the Dana-Farber Cancer Institute [DFCI], resp.) as internal controls. The geometric mean of their C_t_ was used as the endogenous control. Each qPCR reaction was repeated three times. All PCR efficiencies were above 95%. Results of the sequence detection software (version 1.3, Applied Biosystems) were exported as tab-delimited text files and imported into Microsoft Excel for further analysis. The median coefficient of variation (based on calculated quantities) of the duplicate samples was 6%.

### 2.4. GO Enrichment and KEGG Analysis

GO functional enrichment analysis was performed using singular enrichment analysis (SEA) on AgriGO (http://bioinfo.cau.edu.cn/agriGO/) [26]. The MAGA data were used as the background reference, and the hypergeometric test was used for statistical analysis. GO terms could be divided into three categories: biological process, cellular component, and molecular function. All significant GO secondary level terms could be used to generate a flash bar charts showing the overrepresented terms in all three categories. The genes with significant GO categories were subjected to hierarchical clustering analysis using Genesis and Kyoto Encyclopedia of Genes and Genomes (KEGG) analysis.

### 2.5. Statistical Analyses

All results shown in the figures were the mean ± SE of at least three replicates. Significant differences between and among treatments were statistically evaluated by analysis of variance using SAS version 9.0 (SAS Institute Inc., Cary, NC, USA) with statistical significance set at *P* = 0.05. Analysis of correlation coefficients among 226 genes with significantly enriched GO terms was performed using SAS 9.0. The relationship between the TFs and metabolic genes was also studied. Genes that had correlation coefficients (*r*) ≥ 0.7  (*P* ≤ 0.01) or (*r*)≤−0.7  (*P* ≤ 0.01) (especially between genes encoding TFs and metabolism-associated genes) were selected and analyzed (Table 3).

## 3. Results

### 3.1. Seedling Survival Rate

**Figure 1:**
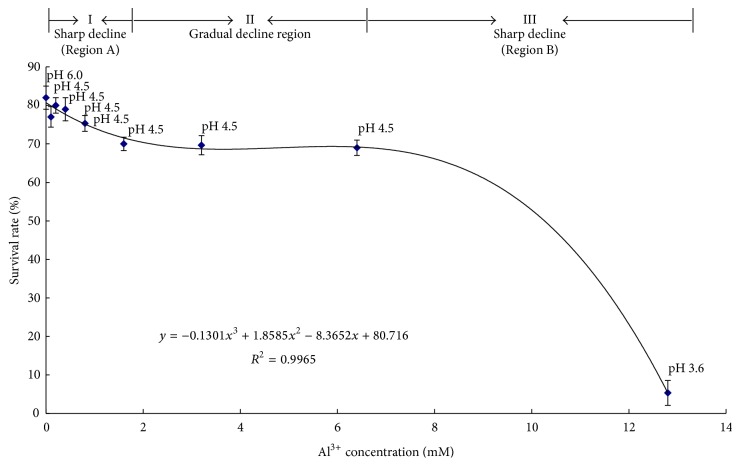
Survival rate of alfalfa under Al stress. The regression equation and the square of correlation coefficient are presented. Based on the regression equation curve, the Al concentration affecting alfalfa germination can be divided into three regions, I of sharp decline (region A); II of gradual decline; III of sudden decline (region B).

The germination of alfalfa declined with increasing concentration of Al ions from 0 mM to 1.6 mM, and the seedling survival rates gradually decreased from 1.6 to 6.4 mM. However, when the concentration of Al ions was increased to 12.8 mM with the pH value of 3.6, the survival rate declined to 5% (*r* ^2^ = 0.997; Figure 1). Based on the regression equation curve shown in Figure 1, the Al concentrations could be divided into three ranges based on its survival rate in alfalfa: concentration of 0–1.6 mM Al caused a sharp decline in the germination and survival rates; 1.6–6.4 mM Al caused a gradual decline; 6.4–12.8 mM Al resulted in most steep decline. Although we tried to represent each stage for our experimental design, it is challengeable to get enough tissue under 6.4–12.8 mM Al. Thus, the treatments of alfalfa seeds germinated in the presence of 0 *μ*M Al^3+^ (pH 6.0), 0 *μ*M Al^3+^ (pH 4.5), 800 *μ*M Al^3+^ (pH 4.5), and 3.2 mM Al^3+^ (pH 4.5) were used for further microarray analysis.

### 3.2. Microarray Data Quality Assessment

**Figure 2:**
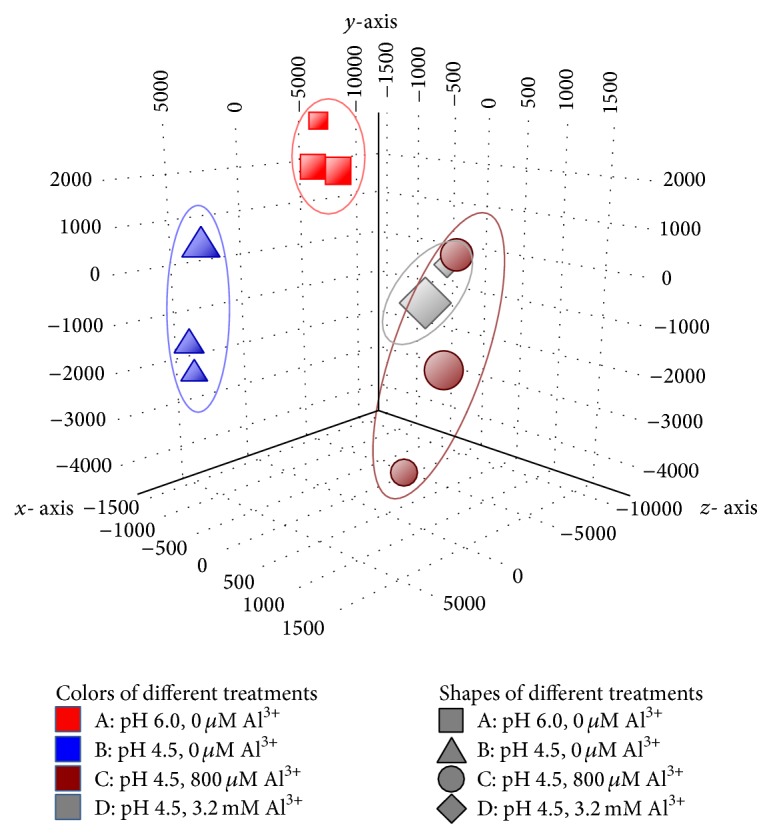
Principle component analysis for microarray data. Principle component analysis of intact genes detected by microarray from samples under different Al treatments for 60 h. The colored graph shows all data points projected in the three-dimensional space formed by three coordinates after rotation. Each data point represents an independent Al treatment, with the red colored square representing samples treated with pH 6.0 and 0 mM Al, the blue triangle representing samples treated with pH 4.5 and 0 mM Al, the brown circle representing samples treated with pH 4.5 and 0.8 mM Al and the gray rhombus representing samples treated with pH 4.5 and 3.2 mM Al. Samples with the same treatment are closely related and can be encircled together.

**Figure 3:**
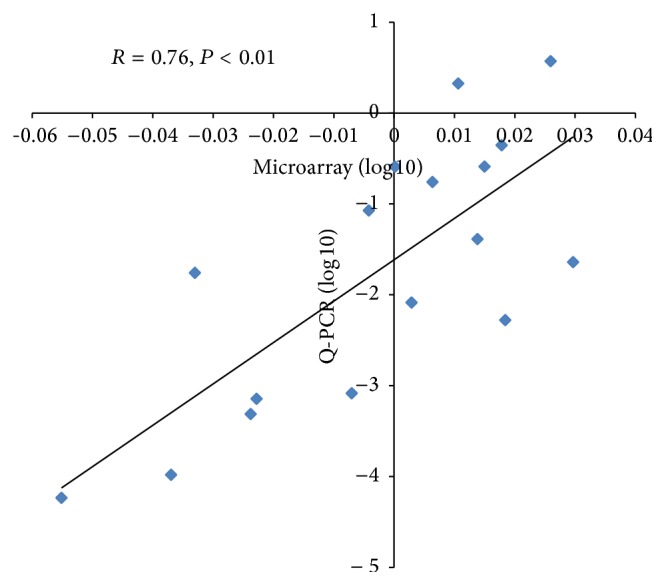
Relationship between microarray and qPCR data. The expression profiles of each gene based on the microarray data and qPCR were log_10_ transformed. The microarray data were plotted against the qPCR data.

The data quality was assessed using two measurements. First, a correlation > 0.96 was obtained among three biological replicates of all of the treatments analyzed. A principal component analysis demonstrated that seedlings treated with or without Al^3+^ at pH 6.0 or 4.5 were distributed in distinct groups (Figure 2). The distinction among data points from different sample treatments validated our experimental pipeline. Second, we validated the expression profiles of 17 genes using qPCR. The results were highly consistent with the data from the microarray analysis (*r* = 0.76; *P* < 0.01) (Figure 3). Taken together, the microarray data obtained in this study were reliable for further study.

### 3.3. Features of the Expressed Genes

Of all the 43,803 probe sets measured by RNA hybridization, 43,651 were found expressed in the seedlings treated with Al at different pH values. The total number was reduced to about 65% of the transcripts after the probe sets with ambiguous signals and those that were not called “present” in at least two replicates were removed (see Table S1 in Supplementary Material available online at http://dx.doi.org/10.1155/2016/2095195). Of these, 4,146 transcripts were up- or downregulated with at least two times of FC and *P* value of 0.05 in the paired *t*-tests (Table 2).

There are 1037 and 912 genes being upregulated in the 800 *μ*M and 3.2 mM Al^3+^ (pH 4.5) groups, respectively, in comparison with the corresponding gene expression levels in the 0 *μ*M Al^3+^ (pH 6.0) group. Among the upregulated genes, 747 were common in both groups (Table 2). 340 and 566 genes were downregulated in the groups treated with 800 *μ*M and 3.2 mM Al^3+^ (pH 4.5), respectively. 242 genes were downregulated in both groups (Table 2). Furthermore, in comparison with the corresponding gene expression levels in the 0 *μ*M Al^3+^ (pH 4.5) group, 1381 and 1893 genes were upregulated in the 800 *μ*M and 3.2 mM Al^3+^ (pH 4.5) groups, respectively, and 1052 were upregulated in both groups (Table 2). 735 and 1,111 genes were downregulated in those two groups, respectively, with 563 genes common in both groups (Table 2). Thus, a total of 3926 genes were found to be either up- or downregulated following exposure to Al^3+^ (FC ≥ 2.0; and *P* < 0.05,* t*-test).

**Figure 4:**
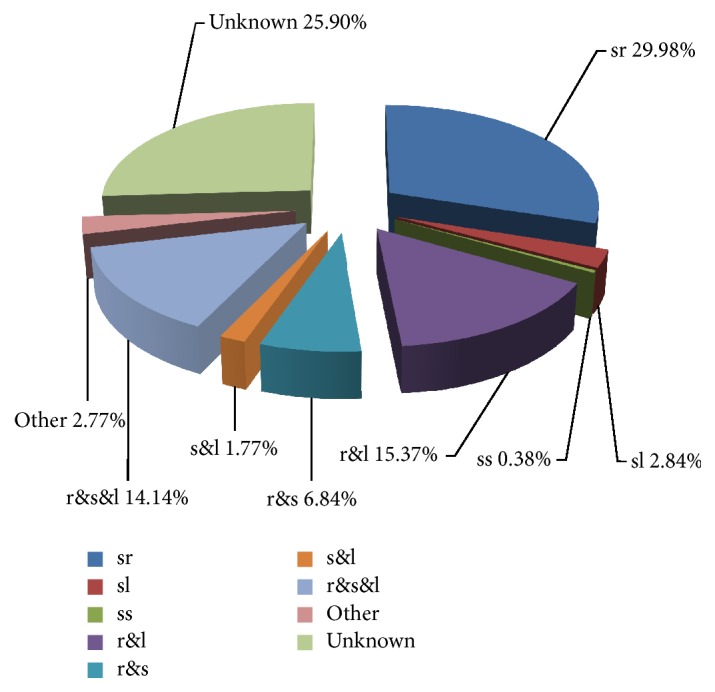
The percentages of expression patterns for differentially expressed genes. The percentages of expression patterns for the 3926 differentially expressed genes were shown in the pie chart. Basically, they were divided into nine types: (1) “sr” meant specifically expressing in roots; (2) “sl” meant specifically expressing in leaves; (3) “ss” meant specifically expressing in stems; (4) “r&l” indicated genes expressed in roots and leaves; (5) “r&s” indicated genes expressed in roots and stems; (6) “s&l” indicated genes expressed in stems and leaves; (7) “r&s&l” indicated genes expressed in roots, stems, and leaves; (8) “other” indicated genes expressed in other plant tissues; (9) “unknown” indicated genes which cannot identify their expression patterns in NCBI databases.

To identify gene expression patterns, the EST sources in NCBI (National Center for Biotechnology Information) databases were used, and the results indicated the expression patterns of the 3926 genes were divided into nine types (Figure 4). Among those nine types, the most noticeable patterns include sr (genes expressed specifically in roots), r&l (genes expressed in roots and leaves), and r&s&l (genes expressed in roots, stems, and leaves), with proportions of 29.98%, 15.37%, and 14.14%, respectively. Genes expressed in root could account for 66.33% of all the genes, which indicated that most gene expressions were induced or inhibited in root under acid-Al stress. Although EST may be incomplete as an indication of gene activity, the comprehensive gene expression patterns did give us hint that gene expressions response to Al stress mainly happened in alfalfa root. In our study, the location of gene expression was shown in a parenthesis following the probe ID. For example, A_27_P051336 (r&s&l) meant the gene A_27_P051336 was expressed in root, stem, and leaf.

We summarized gene lists for response to plant hormone, stress defense, and membrane transporters. A total of 26 genes related to plant hormones (i.e., IAA, ABA, and ethylene) were found (Table S2), in which 9 genes were related to auxin, such as the auxin response protein genes, cationic peroxidase genes with IAA oxidase activity, and auxin conjugate hydrolase gene; 9 genes encoded ethylene responsive transcription factors; 2 genes were related to ABA; 2 genes were related to cytokinin; and 4 genes were related to gibberellin. A total of 28 genes were related to stress defense (Table S3), including an Aluminum sensitive protein, DREB (dehydration responsive element binding) protein, heat shock proteins, LEA (late embryogenesis abundant) proteins, and a universal stress protein. A total of 20 genes were membrane transporters (Table S4), including 8 ABC transporter genes, 6 nitrate transporter genes, peptide transporters, a potassium transporter, a sulfate transporter, a zinc transporter, and a mitochondrial phosphate transporter.

### 3.4. AgriGO Functional Enrichment Analysis of All Differentially Expressed Genes

**Figure 5:**
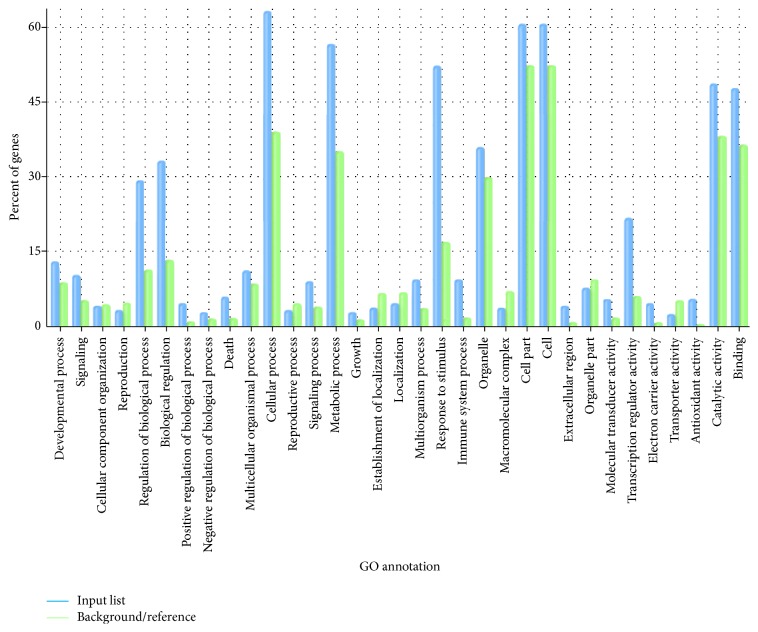
Flash bar chart of overrepresented terms in all three categories. The* y*-axis is the percentage of genes mapped by the term and represents the abundance of the GO term. The percentage for the input list is calculated by the number of genes mapped to the GO term divided by the total number of genes in the input list. The same calculation was applied to the reference list to generate its percentage. These two lists are represented using different colors. The *x*-axis is the definition of the GO terms.

GO functional enrichment was done against the total of 3926 differentially expressed genes. In the biological process category, the percentage of regulation of biological processes (GO:0050789) was 29.5% (12% in MAGA), biological regulation (GO:0065007) was 34% (14% in MAGA), cellular process (GO:0009987) was 63% (41.5% in MAGA), metabolic process (GO:0008152) was 58% (36% in MAGA), and response to stimulus (GO:0050896) was 53% (17.5% in MAGA). In the cellular component category, the percentage of cell (GO:0005623) was 62.3% (52.5% in MAGA). In the molecular function category, the percentage of transcription regulator activity (GO:0030528) was 21% (4% in MAGA) (Figure 5). 79 GO terms were found highly significantly (from the tertiary level to the bottom level) with the threshold of *P* values < 0.001 and false discovery rates (FDR) < 0.05 (Table S5). The most enriched GO terms included metabolic process, response to stimulus, and transcription regulator activity (Figure 5). The highest significance enriched GO terms with *P* values ≤ 5 × 10^−10^ were related to the phenylpropanoid biosynthetic process (GO:0009699), flavonoid metabolic process (GO:0009812), and flavonoid biosynthetic process (GO:0009813), revealing that phenylpropanoid and flavonoid metabolism may be involved in the response to Al stress (Figure S1).

### 3.5. Hierarchical Clustering Analysis

**Figure 6:**
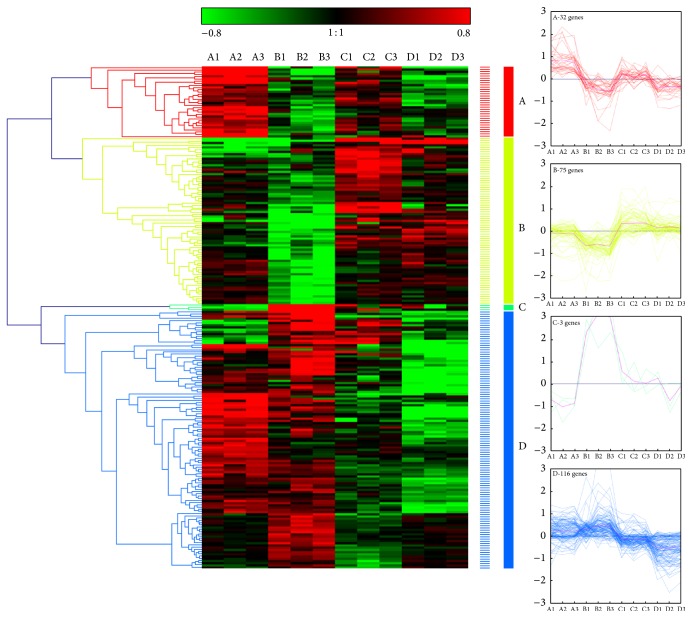
Cluster analysis of the expression profiles. Cluster analysis of the expression profiles of 226 genes in the highly significantly enriched GO terms. Cluster analysis for each group of genes was performed using hierarchical clustering with Genesis 1.7.5 with average linkage and Euclidian distance measurements. Rows represent differentially expressed genes, while columns represent different independent treatments (A, B, C, and D represent the germinated seeds treated with 0 (pH 6.0), 0 (pH 4.5), 0.8 (pH 4.5), and 3.2 (pH 4.5) mM AlCl_3_ solution for 60 h, resp.; each treatment was repeated three times). The color scale shown at the top illustrates the relative expression ratios of genes across all samples. Four line charts of the four groups are shown on the right.

When all of the genes in the 79 significant enrichment GO terms were combined, 226 genes were obtained. The Cluster program generated four different clusters according to the gene expression patterns (Figure 6). Cluster A included 32 genes that were downregulated by acid treatment. The genes in this cluster included those encoding the TFs WRKY 22 (A_27_P136691 (unknown)), MYB-like (A_27_P234867 (unknown)), OCSB factor 1 (A_27_P065811 (sr)), and MYC2 (A_27_P348932 (r&s&l)). Cluster B included 75 genes that were upregulated by Al-acid treatment, including genes encoding the TFs MYB an2 (A_27_P181166 (unknown)) and bHLH120 (A_27_P162931 (sr)) and genes involved in flavonoid biosynthesis (GO:0009813), such as dihydroflavonol 4-reductase (A_27_P258277 (other)) and the HCT (A_27_P263014 (r&s&l)). Cluster C included 3 genes that were upregulated by acid treatment. Cluster D included 116 genes that were downregulated by Al-acid treatment, including genes encoding the TFs WRKY 11 (A_27_P015415 (r&s&l)) and CCCH29-like (A_27_P274912 (r&s&l)) and genes involved in phenylpropanoid metabolism (GO:0009698) terms, such as TT7 (A_27_P042756 (unknown)) and cinnamoyl-CoA reductase (A_27_P045576 (r&s&l)). Comparison of gene expression under acid-Al treatment indicated that gene sets are differentially expressed under each stress treatment.

### 3.6. Kyoto Encyclopedia of Genes and Genomes (KEGG) Analysis of the Pathways

KEGG analysis revealed that phenylpropanoid biosynthesis, phenylalanine metabolism, and starch and sucrose metabolism were the three main pathways in 226 acid-Al-responsive genes (Table S6). Therefore, we focused on the genes involved in these pathways to demonstrate the utility of these data in understanding their specific functions in the response of alfalfa to Al stress.

**Figure 7:**
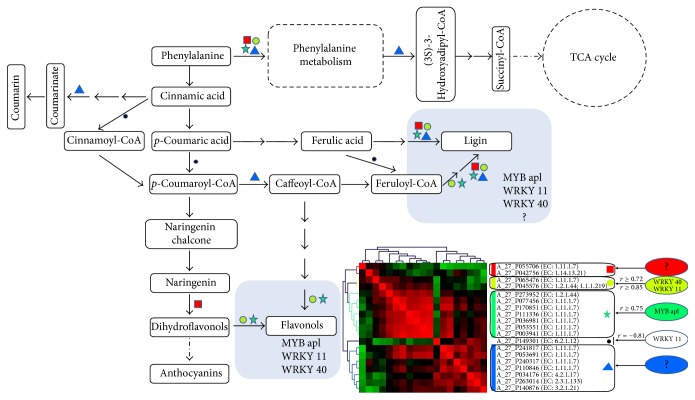
Pathways in response to acid-Al ions based on KEGG analysis. Overview of the combinations of the phenylpropanoid biosynthesis, phenylalanine metabolism, and flavonoid biosynthesis pathways in response to acid-Al ions based on KEGG analysis. Nineteen genes were differentially expressed in response to acid-Al ions. Hierarchical clustering of the results of the correlation coefficient analysis was shown in the lower right corner. The divided groups were marked by different shapes and color icons, which were also marked in the metabolic pathway. High positive correlation coefficient with genes in the divided groups were shown near the image of the hierarchical clustering analysis and also marked in the light shadow zone of the metabolic pathway.

Because the phenylpropanoid biosynthesis, phenylalanine metabolism, and flavonoid biosynthesis pathways were closely linked in plant metabolism, they were combined together in this analysis. The results showed 17 up- or downregulated genes were involved in phenylpropanoid biosynthesis. They encoded 6 key enzymes. Furthermore, 14 differentially expressed genes encoding 3 enzymes were involved in phenylalanine metabolism, and 4 genes encoding 3 enzymes were involved in flavonoid biosynthesis. Totally, 19 genes encoding 9 enzymes were involved in those three linked pathways (Figure S2). 11 genes encoded peroxidases, which were related to reactive oxygen species (ROS) scavenging and lignin synthesis. Based on the correlation coefficient analysis, many TFs, such as MYB305, MYB ap1, WRKY 40, and WRKY 11, were found to have high positive correlation coefficients with genes in this metabolic pathway (Table 3), indicating that these MYB family genes and WRKY family genes and others genes showed in Table 3 may be related to metabolic pathway regulation. Specifically, MYB apl, WRKY 11, and WRKY 40 had high positive correlation coefficients with genes related to ROS scavenging and lignin and flavonoid synthesis (*r* > 0.7, *P* < 0.01; Figure 7).

Finally, 12 genes encoding 7 enzymes were involved in starch and sucrose metabolism. Most enzymes can catalyze the hydrolysis of disaccharides to monosaccharides (Figure S3). However, we failed to find TFs with high positive correlation coefficients with genes involved in starch and sucrose metabolism.

## 4. Discussion

### 4.1. Global Gene Expression Analysis of Alfalfa Exposed to Acid and High Concentrations of Al Ions

Global gene expression analysis using the Agilent gene expression microarray revealed the expression of 3926 genes that were changed by acid and Al ions, in which 66.33% can be detected in root including those specifically expressing in root which accounted for 29.98% of all the genes. Many genes related to phytohormones and ROS-metabolism were identified in the gene expression analysis. Potters et al. [28] hypothesized that Al toxicity can induce SIMR (stress-induced morphogenic response). Interestingly, phytohormones and ROS production are the main regulatory interactions controlling stress-induced SIMR of plants [28]. Thus, genes related to phytohormones and ROS-metabolism may be involved in SIMR following exposure to Al. In this study, the expression of 9 genes related to auxin and 9 genes related to ethylene were found to be up- or downregulated, revealing for the response of auxin and the ethylene to Al toxicity. Auxin and ethylene were found to play an important role in regulating Aluminum-induced inhibition of root growth [6, 29, 30]. Sun et al. [2] found that Al-induced ethylene may act as a signal to alter auxin distribution in roots, thereby inhibiting root elongation. Twelve genes encoding peroxidase (GO:0004601) could eliminate ROS production, leading to the plant morphological changes induced by Al.

A total of 20 genes encoding transporters were found in our Agilent gene expression microarray analysis, in which 8 genes encoded ABC transporters. ABC transporters contain an ATP-binding cassette (ABC) and participate directly in the transport of a wide range of molecules across membranes [31]. ABC transporters also participated in transporting phytohormones such as auxin and abscisic acid and were involved in the detoxification of toxic minerals, such as cadmium (Cd), arsenic (As), and Aluminum (Al) [32].

The AgriGO and KEGG analyses revealed 19 genes encoding 9 key enzymes involved in phenylpropanoid biosynthesis, phenylalanine metabolism, and flavonoid biosynthesis metabolism after the addition of acidic pH and high Al^3+^ concentrations (Figure S2). The activation of these pathways indicated that they were associated with response to Aluminum stress. Phenylpropanoid compounds produced by the phenylpropanoid and flavonoid biosynthesis metabolism pathways could play a variety of roles in plant defense [33, 34]. The phenylpropanoid compound formed complexes with Al ions and led to internal Al detoxification in Al-accumulating species [11, 12]. Kováčik et al. [35] suggested that phenolic compounds might also affect shoot Al uptake. The POD (EC 1.11.1.7) in the phenylpropanoid pathway can scavenge ROS produced by Al stress [36, 37]. Furthermore, the lignin produced by phenylpropanoid biosynthesis would affect the cell wall composition and the SIMR under Al stress [28, 38].

A total of 14 genes encoding 3 enzymes (POD (EC 1.11.1.7), enoyl-CoA hydratase, and 4-coumarate-CoA ligase) that took part in the phenylalanine metabolism pathway were affected by Al. The gene expression changes induced by Al would affect the synthesis of succinyl-CoA and in turn influenced OA synthesis in the TCA (tricarboxylic acid) cycle. An et al. [10] investigated that Al could affect the synthesis of OAs by influencing the TCA cycle, and the foliar application of succinic acid could increase the accumulation of organic acids (including oxalic acid, malic acid, citric acid, and succinic acid) and alleviate Al toxicity.

### 4.2. TFs Regulating the Metabolic Pathways Were Activated by Acid-Al Stress

Although the phenylpropanoid and flavonoid biosynthesis pathways in plants have been extensively studied [39], limited information is available for gene regulations involved in Al stress. Many studies have discussed the transcriptional regulation of the phenylpropanoid and flavonoid biosynthesis pathways [40]. One of genes regulating the phenylpropanoid pathway was AmMYB308, which was found in* Antirrhinum majus *[41]. AmMYB308 repressed several phenylpropanoid pathway genes when overexpressed in transgenic tobacco. Jin et al. [42] demonstrated that AtMYB4 could inhibit the general phenylpropanoid pathway in* Arabidopsis*. This regulator was the first example of an MYB protein that functioned as a transcriptional repressor in the phenylpropanoid pathway in* Arabidopsis* [43]. Hichri et al. [40] proposed a model in which the phenylpropanoid and flavonoid biosynthesis pathways were controlled by a complex of the MYB, basic helix-loop-helix (bHLH), and WD40 proteins.

In this study, we used correlation coefficient analysis to study the possible TFs regulating the metabolic pathways activated by Al stress. MYB family genes such as MYB 305, MYB an2, and MYB apl were found to have high positive correlation coefficients with genes involved in the phenylpropanoid and flavonoid biosynthesis pathways (*r* ≥ 0.7, *P* ≤ 0.01; Table 3). Furthermore, MYB 305 was found to have a significant negative correlation with TT7 (flavonoid 3′-monooxygenase, A_27_P042756 (unknown); *r* = −0.73 and *P* < 0.01), revealing that this gene may suppress the expression of TT7. In* Arabidopsis*, the MYB family gene PAP1, which was homologous to MYB 305, could suppress the expression of TT7; this result was consistent with our data [44]. Many previous reports have suggested that bHLH may be a cofactor of MYB [40, 45, 46]. In our study, we found that the bHLH gene bHLH35 had a high positive correlation coefficient with many genes in the phenylpropanoid and flavonoid biosynthesis pathways (Table 3). The genes of the WRKY family (WRKY 40 and WRKY 11) were also found to have high positive correlation coefficients with genes related to lignin and flavonoid synthesis (*r* > 0.7, *P* < 0.01), indicating the possible regulatory roles of WRKY 40 and WRKY 11 in the phenylpropanoid and flavonoid biosynthesis pathways. However, no study has discussed the relationship between the WRKY family and the phenylpropanoid biosynthesis pathway to date.

## 5. Conclusion

Global gene expression analysis showed that acid-Al could significantly affect 3,926 gene expression. The fact of 66.33% of differentially expressed genes from roots verified that the primary target of acid-Al toxicity in plants was the root. GO enrichment and KEGG study indicated that the phenylpropanoid, flavonoid biosynthesis, and transcription factors of MYB and WRKY families were mainly involved in the response to acid-Al stress in alfalfa. Understanding the key regulatory genes and pathways would be advantageous to produce a better crop yield on acid soils and Al stress not only in alfalfa but also in other crops.

## Supplementary Material

The supplementary material includes three figures (Figure S1, Hierarchical tree graph of overrepresented GO terms; Figure S2, Overview of the combinations of phenylpropanoid biosynthesis, phenylalanine metabolism, and flavonoid biosynthesis metabolism pathway; Figure S3, The starch and sucrose metabolism pathway) and six tables (Table S1, The number of probes detected in the microarrays; Table S2, Genes up- or down-regulated following exposure to acid-Al related to plant hormones; Table S3, Genes up- or down-regulated following exposure to acid-Al related to stress defense; Table S4, Genes up- or down-regulated following exposure to acid-Al are membrane transporters; Table S5, Detail information of high significant enrichment GO terms list; Table S6, List of the metabolic pathways of 226 genes which belonged to significant enrichment GO terms).

## Figures and Tables

**Table 1:** Primer sequences used for qPCR.

Probe Name	Description	Forward sequence	Reverse sequence	Accession number
A_27_P077456	Peroxidase-456	5′-AGGAAATCTAAGGTGGCAACTG-3′	5′-TTTAGGTAAGCCAGGAATGTGG-3′	TC192119 (DFCI)
A_27_P091936	Resistance protein-936	5′-ATCGTGGAATGGGAAAGACAAC-3′	5′-CCAGGACCAAACCAATCAAGT-3′	TC181653 (DFCI)
A_27_P015415	WRKY 11	5′-TCATTTCCTCTGGCAAGCCT-3′	5′-TCAGCGACCTTTGAACTTATCG-3′	TC175297 (DFCI)
A_27_P274912	CCCH29-LIKE	5′-CAAGAGGGAAGTAGATGAGAAGGA-3′	5′-CAACAGCACAATGAAGAGCAG-3′	TC199223 (DFCI)
A_27_P133981	Myb-like	5′-AGAGGACAATGGGAAAGAAGAC-3′	5′-CAGCACTTGATGCCTAAGACA-3′	TC183988 (DFCI)
A_27_P155316	Nbs-containing resistance-like protein	5′-AACGCTCTGAACAACGAGGA-3′	5′-CACCGAAATCACACTCCGAG-3′	XM_003588950
A_27_P055706	Peroxiredoxin-chloroplastic-like	5′-GAATCCACCTTCTCCTACCTCG-3′	5′-CTTCAAATCCTCCTTCCACGC-3′	TC185056 (DFCI)
A_27_P181166	MYB an2	5′-GGACATACGAGGAAGACAACTTAC-3′	5′-CCTTCCAGCAATCAATGACCAT-3′	XM_003621524
A_27_P050191	Pathogenesis-related protein 4	5′-TGGTTACGGGATGTCTCAAGG-3′	5′-TTTGGTGTTGGTGTTGGTGC-3′	TC183745 (DFCI)
A_27_P162931	bHLH120	5′-TCAACCACCACCAACATCAC-3′	5′-TGAAGGGTAGCCATTTCTTGTC-3′	TC190718 (DFCI)
A_27_P111336	Peroxidase 43-like	5′-GTCCAGGAGTGGTTTCTTGTG-3′	5′-CTGTGAGACCCTTGTTTAGGAAC-3′	TC190043 (DFCI)
A_27_P036981	Peroxidase 12-like	5′-TTCCTCTGTTCTGGCTAATGGT-3′	5′-AGCACCTGAAAGGGCAACTA-3′	TC196923 (DFCI)
A_27_P104616	bZIP60	5′-ATCCTTCTGTTTCCGTCGCA-3′	5′-TCCCTGTTCCTCATCTGCCT-3′	TC180070 (DFCI)
A_27_P136211	WRKY 22	5′-CCCTAAAGAGCCTGAACAAGTC-3′	5′-GCTTCGTGGATAAGGTGAACC-3′	TC196399 (DFCI)
A_27_P123906	DREF1	5′-CCTTCCTATTCCAGCAACTTCC-3′	5′-CCTGTTCATCAACTTCCACACA-3′	TC182024 (DFCI)
A_27_P045576	Anthocyanidin reductase-like	5′-CATTTACCGACCCTGCTGGT-3′	5′-TCTCTGCCCTCATCTTGCCT-3′	TC186981 (DFCI)
A_27_P060501	P450 83b1-like	5′-GTTTGTAGGACTGCGTTCGG-3′	5′-TTCGTGTGGAGGCAACTTCT-3′	TC173827 (DFCI)
N	EF-*α*	5′-GCACCAGTGCTCGATTGC-3′	5′-TCGCCTGTCAATCTTGGTAACAA-3′	XM_003618727
N	18S ribosomal RNA gene	5′-TCAGAGGATGGCGACGAAG-3′	5′-CCGTTGCCGAGAGTCATTCT-3′	DQ311983
N	Ubiquitin	5′-CTCACTGGAAAGACAATCACCC-3′	5′-GAAGTCGCAACACAAGATGGA-3′	TC174254 (DFCI)

**Table 2:** Differentially expressed genes across all treatments.

	B versus A	C versus A	D versus A	C versus B	D versus B	D versus C
Downregulated	1126	340	566	735	1111	595
Upregulated	1379	1037	912	1381	1893	304
Total	4146					

All of the genes mapped to the reference sequence and genome sequences were examined for differences in expression across the different libraries. Numbers of differentially expressed genes were analyzed across sense transcripts using a threshold value FC ≥ 2. A, B, C, and D represent germinated seeds treated with 0 (pH 6.0), 0 (pH 4.5), 0.8 (pH 4.5), and 3.2 (pH 4.5) mM AlCl_3_ solution for 60 h, respectively.

**Table 3:** Correlation coefficient analysis of TFs with metabolic pathway genes.

TF	Probe	Genes having highly positive correlation coefficients with TF	Genes having significant negative correlation with TF
MYB 305	A_27_P172741 (unknown)	A_27_P036981 (sr) (peroxidase; EC: 1.11.1.7), *r* = 0.91, *P* < 0.001	A_27_P042756 (unknown) (flavonoid 3′-monooxygenase; EC: 1.14.13.21), *r* = −0.73, *P* = 0.006
		A_27_P053551 (r&s) (peroxidase; EC: 1.11.1.7), *r* = 0.88, *P* < 0.001	
		A_27_P111336 (unknown) (peroxidase; EC: 1.11.1.7), *r* = 0.81, *P* = 0.0013	
		A_27_P003941 (sr) (peroxidase; EC: 1.11.1.7), *r* = 0.81, *P* = 0.0013	

MYB apl	A_27_P129206 (unknown)	A_27_P077456 (unknown) (peroxidase; EC: 1.11.1.7), *r* = 0.75, *P* = 0.005	
		A_27_P036981 (sr) (peroxidase; EC: 1.11.1.7), *r* = 0.87, *P* < 0.001	
		A_27_P241817 (sr) (peroxidase; EC: 1.11.1.7), *r* = 0.88, *P* < 0.001	
		A_27_P111336 (unknown) (peroxidase; EC: 1.11.1.7), *r* = 0.77, *P* = 0.003	
		A_27_P170851 (unknown) (peroxidase; EC: 1.11.1.7), *r* = 0.79, *P* = 0.002	
		A_27_P053691 (unknown) (peroxidase; EC: 1.11.1.7), *r* = 0.80, *P* = 0.0015	
		A_27_P003941 (sr) (peroxidase; EC: 1.11.1.7), *r* = 0.86, *P* < 0.001	
		A_27_P273952 (r&s&l) (cinnamoyl-CoA reductase; dihydrokaempferol 4-reductase; EC: 1.2.1.44; 1.1.1.219), *r* = 0.72, *P* = 0.008	

MYB an2	A_27_P181166 (unknown)	A_27_P241317 (r&s&l) (peroxidase; EC: 1.11.1.7), *r* = 0.70, *P* = 0.01	

MYB-like	A_27_P133981 (sr)	A_27_P053551 (r&s) (peroxidase; EC: 1.11.1.7), *r* = 0.70, *P* = 0.01	A_27_P149301 (unknown) (4-coumarate-CoA ligase; EC: 6.2.1.12), *r* = −0.76, *P* = 0.004
		A_27_P111336 (unknown) (peroxidase; EC: 1.11.1.7), *r* = 0.83, *P* < 0.001	
		A_27_P273952 (r&s&l) (cinnamoyl-CoA reductase; dihydrokaempferol 4-reductase; EC: 1.2.1.44; 1.1.1.219), *r* = 0.80, *P* = 0.0015	
		A_27_P045576 (r&s&l) (cinnamoyl-CoA reducatase; dihydrokaempferol 4-reducatse; EC: 1.2.1.44; 1.1.1.219) *r* = 0.87, *P* < 0.001	

MYC2	A_27_P057826 (r&s&l)	A_27_P273952 (r&s&l) (cinnamoyl-CoA reductase; dihydrokaempferol 4-reductase; EC: 1.2.1.44; 1.1.1.219), *r* = 0.76, *P* = 0.003	
		A_27_P045576 (r&s&l) (cinnamoyl-CoA reductase; dihydrokaempferol 4-reductase; EC: 1.2.1.44; 1.1.1.219) *r* = 0.83, *P* < 0.001	

MYC2-like	A_27_P348932 (r&s&l)	A_27_P273952 (r&s&l) (cinnamoyl-CoA reductase; dihydrokaempferol 4-reductase; EC: 1.2.1.44; 1.1.1.219), *r* = 0.95, *P* < 0.001	

OCS TF	A_27_P065811 (sr)	A_27_P077456 (unknown) (peroxidase; EC: 1.11.1.7), *r* = 0.89, *P* < 0.001	A_27_P149301 (unknown) (4-coumarate-CoA ligase; EC: 6.2.1.12), *r* = −0.72, *P* = 0.008
		A_27_P036981 (sr) (peroxidase; EC: 1.11.1.7), *r* = 0.72, *P* = 0.008	
		A_27_P053551 (r&s) (peroxidase; EC: 1.11.1.7), *r* = 0.81, *P* = 0.0013	
		A_27_P111336 (unknown) (peroxidase; EC: 1.11.1.7), *r* = 0.82, *P* = 0.001	
		A_27_P170851 (unknown) (peroxidase; EC: 1.11.1.7), *r* = 0.79, *P* = 0.002	
		A_27_P003941 (sr) (peroxidase; EC: 1.11.1.7), *r* = 0.83, *P* < 0.001	
		A_27_P273952 (r&s&l) (cinnamoyl-CoA reductase; dihydrokaempferol 4-reductase; EC: 1.2.1.44; 1.1.1.219), *r* = 0.78, *P* = 0.0025	

WRKY 40	A_27_P031021 (sr)	A_27_P065476 (sr) (peroxidase; EC: 1.11.1.7), *r* = 0.77, *P* = 0.003	
		A_27_P045576 (r&s&l) (cinnamoyl-CoA reductase; dihydrokaempferol 4-reductase; EC: 1.2.1.44; 1.1.1.219) *r* = 0.89, *P* < 0.001	
		A_27_P273952 (r&s&l) (cinnamoyl-CoA reductase; dihydrokaempferol 4-reductase; EC: 1.2.1.44; 1.1.1.219), *r* = 0.72, *P* = 0.008	

WRKY 11	A_27_P015415 (r&s&l)	A_27_P065476 (sr) (peroxidase; EC: 1.11.1.7), *r* = 0.85, *P* < 0.001 A_27_P045576 (r&s&l) (cinnamoyl-CoA reductase; dihydrokaempferol 4-reductase; EC: 1.2.1.44; 1.1.1.219) *r* = 0.83, *P* < 0.001	A_27_P149301 (unknown) (4-coumarate-CoA ligase; EC: 6.2.1.12), *r* = −0.81, *P* = 0.001
			A_27_P263014 (r&s&l) (Shikimate O-hydroxycinnamoyl transferase; EC: 2.3.1.133), *r* = −0.71, *P* = 0.009
			A_27_P034176 (r&s) (3-hydroxyisobutyryl-hydrolase 1; EC: 4.2.1.17), *r* = −0.85, *P* < 0.001

ERF13	A_27_P008251 (sr)	A_27_P077456 (unknown) (peroxidase; EC: 1.11.1.7), *r* = 0.73, *P* = 0.007	
		A_27_P111336 (unknown) (peroxidase; EC: 1.11.1.7), *r* = 0.79, *P* = 0.002	
		A_27_P065476 (sr) (peroxidase; EC: 1.11.1.7), *r* = 0.71, *P* = 0.0096	
		A_27_P273952 (r&s&l) (cinnamoyl-CoA reductase; dihydrokaempferol 4-reductase; EC: 1.2.1.44; 1.1.1.219), *r* = 0.88, *P* < 0.001	
		A_27_P045576 (r&s&l) (cinnamoyl-CoA reductase; dihydrokaempferol 4-reductase; EC: 1.2.1.44; 1.1.1.219), *r* = 0.82, *P* = 0.001	

bHLH35	A_27_P048601 (unknown)	A_27_P036981 (sr) (peroxidase; EC: 1.11.1.7), *r* = 0.76, *P* = 0.004	A_27_P149301 (unknown) (4-coumarate-CoA ligase; EC: 6.2.1.12), *r* = −0.70, *P* = 0.01
		A_27_P053551 (r&s) (peroxidase; EC: 1.11.1.7), *r* = 0.76, *P* = 0.004	
		A_27_P111336 (unknown) (peroxidase; EC: 1.11.1.7), *r* = 0.87, *P* < 0.001	
		A_27_P273952 (r&s&l) (cinnamoyl-CoA reductase; dihydrokaempferol 4-reductase; EC: 1.2.1.44; 1.1.1.219), *r* = 0.83, *P* < 0.001	
		A_27_P045576 (r&s&l) (cinnamoyl-CoA reductase; dihydrokaempferol 4-reductase; EC: 1.2.1.44; 1.1.1.219) *r* = 0.81, *P* = 0.001	

CCCH 29	A_27_P274912 (r&s&l)	A_27_P045576 (r&s&l) (cinnamoyl-CoA reductase; dihydrokaempferol 4-reductase; EC: 1.2.1.44; 1.1.1.219) *r* = 0.81, *P* = 0.001	A_27_P149301 (unknown) (4-coumarate-CoA ligase; EC: 6.2.1.12), *r* = −0.84, *P* < 0.001
			A_27_P034176 (r&s) (3-hydroxyisobutyryl-hydrolase 1; EC: 4.2.1.17), *r* = −0.81, *P* = 0.001
