# Supplementary material for: Gene Expression Analysis of Alfalfa Seedlings Response to Acid-Aluminum
Source: Int J Genomics. 2016 Dec 15;2016:2095195. doi: 10.1155/2016/2095195 (PMC5198156; doi:10.1155/2016/2095195)
Supplement: Supplementary file 1 — The supplementary material includes three figures (Figure S1, Hierarchical tree graph of overrepresented GO terms; Figure S2, Overview of the combinations of phenylpropanoid biosynthesis, phenylalanine metabolism, and flavonoid biosynthesis metabolism pathway; Figure S3, The starch and sucrose metabolism pathway) and six tables (Table S1, The number of probes detected in the microarrays; Table S2, Genes up- or down-regulated following exposure to acid-Al related to plant hormones; Table S3, Genes up- or down-regulated following exposure to acid-Al related to stress defense; Table S4, Genes up- or down-regulated following exposure to acid-Al are membrane transporters; Table S5, Detail information of high significant enrichment GO terms list; Table S6, List of the metabolic pathways of 226 genes which belonged to significant enrichment GO terms). [file 2095195.f1.pdf]

supplementary figures

Figure S1. Hierarchical tree graph of overrepresented GO terms

Hierarchical tree graph of overrepresented GO terms in biological process category was generated by SEA. Boxes in the graph represent GO terms labeled by their GO ID, term definition and statistical information. The significant term (adjusted P ≤ 0.05) were marked with color, while non-significant terms were shown as white boxes. The diagram, the degree of color saturation of a box was positively correlated to the enrichment level of the term. Solid, dashed, and dotted lines represent two, one and zero enriched terms at both ends connected by the line, respectively. The rank direction of the graph was set to from top to bottom.

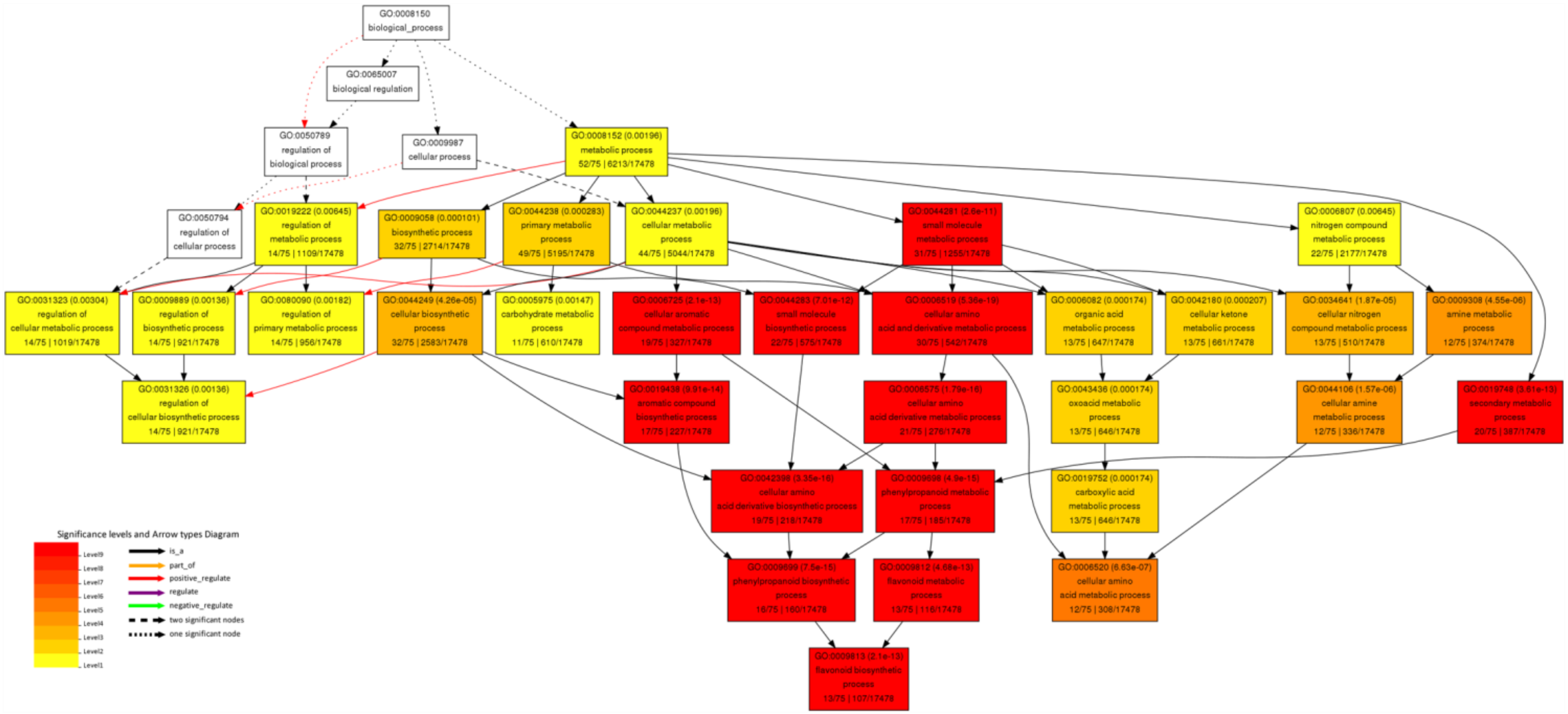

Figure.S2. Overview of the combinations of phenylpropanoid biosynthesis, phenylalanine metabolism, and flavonoid biosynthesis metabolism pathway in response to high concentrations of acid-Al treatment were shown based on KEGG analysis.

The box on the top of the figure shows the name and EC number of enzymes changed by Al ions treatment. The expression profiles of each enzymes based on microarray data were shown around the metabolism pathway.

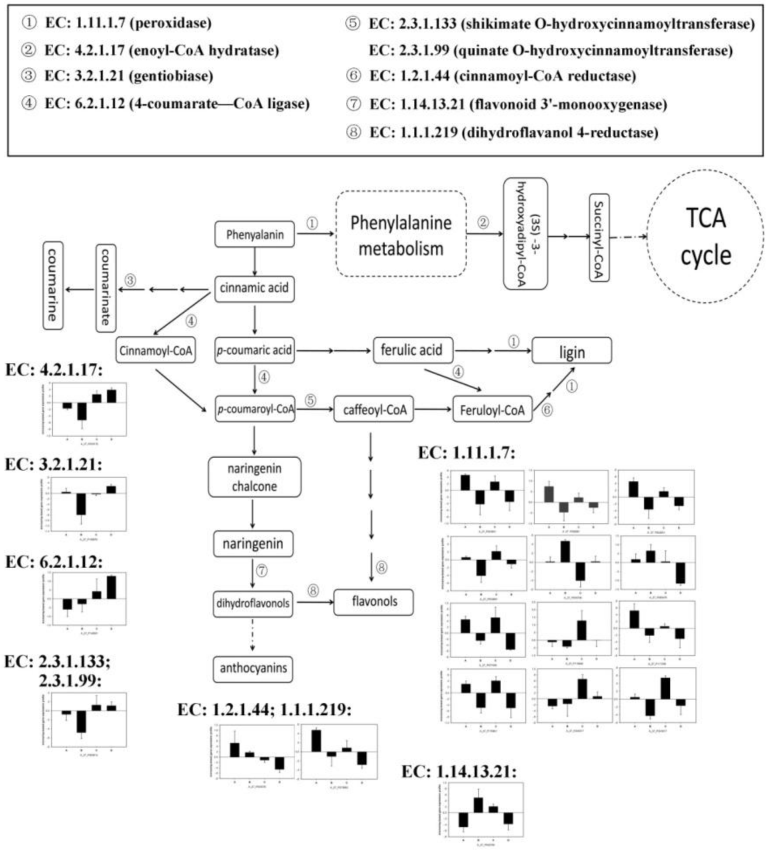

Figure.S3. The starch and sucrose metabolism pathway in response to high concentrations of acid-Al treatment were shown based on KEGG analysis.

The box on the top of the figure shows the name and EC number of enzymes changed by Al ions treatment. The expression profiles of each enzymes based on microarray data were shown around the metabolism pathway.

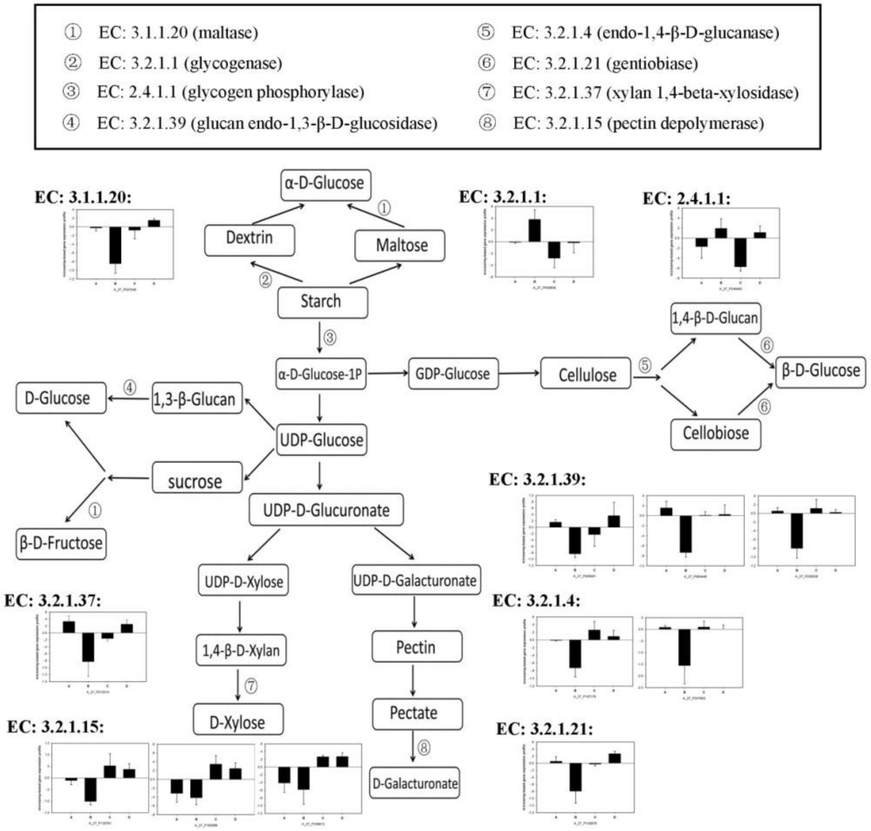

supplementary tables

Table S1

The number of probes detected in the microarrays.

Total of 43，651 were detected after removing probe sets with ambiguous signals and those that were not called “present” in at least two replicates expression. A, B, C, and D represent the samples which were collected after germination treated with 0 (pH6.0), 0 (pH4.5), 0.8 (pH4.5), and 3.2 (pH4.5) mM $AlCl_3$ solution 60h, spectively. Each treatment was repeat three times and the number of probes detected and the rate of detected probe were shown in the table.

	repeat1	rate1	repeat2	rate2	repeat3	rate3
A	26740	0.612586	27220	0.623583	27875	0.638588
B	29867	0.684223	29566	0.677327	30220	0.692309
C	28742	0.65845	27807	0.63703	28721	0.657969
D	30724	0.703856	25537	0.855027	29080	0.666193
TOTAL			43651			
average rate			0.653095			

Table S2

Genes up- or down-regulated following exposure to acid-Al related to plant hormones(FC ≥ 2.0; P < 0.05, *t* test).

A, B, C, and D represent the samples which were collected after germination treated with 0 (pH6.0), 0 (pH4.5), 0.8 (pH4.5), and 3.2 (pH4.5) mM $AlCl_3$ solution 60h, spectively.

Gene	Flod Chang	Description
**C vs A up**		
A_27_P287213(other)	2.4789245	Auxin-responsive aux/iaa gene family member---Rep: Chromosome chr11 scaffold_13, whole genome shotgun sequence - Vitis vinifera (Grape), partial (40%) [TC196720]
A_27_P051336(r&s&l)	1.9902297	Auxin response factor-like protein---AC2438 NOLLY Medicago truncatula cDNA 5', mRNA sequence [DY616830]
A_27_P361612(unknown)	1.6151618	Rep: Auxin induced proline rich protein - Medicago sativa (Alfalfa), partial (27%) [TC199957]
A_27_P356147(unknown)	1.568109	Rep: Auxin conjugate hydrolase - Medicago truncatula (Barrel medic), partial (19%) [TC197111]
A_27_P206839(r&s&l)	2.5553894	Peroxidase---Rep: Cationic peroxidase - Cicer arietinum (Chickpea) (Garbanzo), partial (32%) [TC196397]
A_27_P057771(r&s&l)	1.599031	Rep: Cationic peroxidase 2 precursor - Arachis hypogaea (Peanut), partial (93%) [TC180791]
**D vs A up**		
A_27_P051336(r&s&l)	2.38706	Auxin response factor-like protein ---AC2438 NOLLY Medicago truncatula cDNA 5', mRNA sequence [DY616830]
A_27_P287213(other)	2.0311565	Auxin-responsive aux/iaa gene family member---Rep: Chromosome chr11 scaffold_13, whole genome shotgun sequence - Vitis vinifera (Grape), partial (40%) [TC196720]
A_27_P361612(unknown)	1.8596011	Rep: Auxin induced proline rich protein - Medicago sativa (Alfalfa), partial (27%) [TC199957]
A_27_P103166(r&s)	2.0577576	Ethylene responsive transcription factor 2b---Rep: ERF-like protein - Cucumis melo (Muskmelon), partial (22%) [TC175128]
A_27_P206839(r&s&l)	3.6565163	Rep: Cationic peroxidase - Cicer arietinum (Chickpea) (Garbanzo), partial (32%) [TC196397]
A_27_P057771(r&s&l)	1.5778235	Rep: Cationic peroxidase 2 precursor - Arachis hypogaea (Peanut), partial (93%) [TC180791]
**D vs B up**		

A_27_P197501(r&l)	2.000976	Auxin-induced protein 5NG4 ---MTYE075TF JCVI-MT1 Medicago truncatula cDNA 5', mRNA sequence [EV259839]
A_27_P356147(unknown)	1.5459893	Rep: Auxin conjugate hydrolase - Medicago truncatula (Barrel medic), partial (19%) [TC197111]
A_27_P352047(r&s&l)	2.1281028	Ethylene insensitive 3-like protein---Medicago truncatula clone MTYFL_FM_FN_FO1G-P-3 unknown mRNA [BT052922]
A_27_P351782(r&s)	2.8180926	Ethylene-responsive transcription factor RAP2-6 ---EST508515 HOGA Medicago truncatula cDNA clone pHOGA-15I17 5' end, mRNA sequence [BG646896]
A_27_P206839(r&s&l)	2.8149004	Peroxidase---Rep: Cationic peroxidase - Cicer arietinum (Chickpea) (Garbanzo), partial (32%) [TC196397]
A_27_P244732(unknown)	1.7432483	Rep: Gibberellin oxidase-like protein - Arabidopsis thaliana (Mouse-ear cress), partial (38%) [TC196087]
A_27_P029386(r&s&l)	2.1893783	ABSCISIC ACID-INSENSITIVE 5-like protein---MTYEX19TF JCVI-MT1 Medicago truncatula cDNA 5', mRNA sequence [EV262556]
**C vs B up**		
A_27_P102891(r&l)	2.0449076	Auxin-induced protein 5NG4---Rep: Chromosome undetermined scaffold_125, whole genome shotgun sequence - Vitis vinifera (Grape), partial (81%) [TC173040]
A_27_P287213(other)	2.088349	Auxin-responsive aux/iaa gene family member---Rep: Chromosome chr11 scaffold_13, whole genome shotgun sequence - Vitis vinifera (Grape), partial (40%) [TC196720]
A_27_P356147(unknown)	1.6829602	Rep: Auxin conjugate hydrolase - Medicago truncatula (Barrel medic), partial (19%) [TC197111]
A_27_P063746(r&s&l)	2.0049205	Ethylene-responsive transcription factor RAP2-4---EST529326 GPOD Medicago truncatula cDNA clone pGPOD-1G16 5' end, mRNA sequence [BI307916]
A_27_P117876(sr)	1.9857155	Rep: Cationic peroxidase 1 precursor - Arachis hypogaea (Peanut), partial (64%) [TC189555]
A_27_P029386(r&s&l)	2.2444928	ABSCISIC ACID-INSENSITIVE 5-like protein---MTYEX19TF JCVI-MT1 Medicago truncatula cDNA 5', mRNA sequence [EV262556]
**D vs B down**		
A_27_P056166(r&s)	-3.841897	Ethylene-responsive transcription factor 1A---EST484069 GVN Medicago truncatula cDNA clone pGVN-69I8 5' end, mRNA sequence [BG582327]

A_27_P087211(r&l)	-2.132371	Ethylene-responsive transcription factor---Rep: EREBP-3 homolog - Stylosanthes hamata (Caribbean stylo), partial (46%) [TC176356]
A_27_P064221(r&s)	-2.6987448	PREDICTED: ethylene-responsive transcription factor ERF054-like [Glycine max]---NF008B02ST1F1000 Developing stem Medicago truncatula cDNA clone NF008B02ST 5', mRNA sequence [AW688479]
A_27_P273002(r&l)	-2.1938486	weakly similar to XP_003594598.1 Ethylene-regulated transcript 2 (ERT2) [Medicago truncatula]---EST611141 KVKC Medicago truncatula cDNA clone pKVKC-7D10, mRNA sequence [BQ165272]
A_27_P066811(r&s&l)	-2.078184	Chitin-inducible gibberellin-responsive protein---Rep: Chromosome undetermined scaffold_610, whole genome shotgun sequence - Vitis vinifera (Grape), partial (56%) [TC177482]
A_27_P079281(r&s&l)	-2.187022	Chitin 角素-inducible gibberellin-responsive protein---MTYEA44TF JCVI-MT1 Medicago truncatula cDNA 5', mRNA sequence [EV260675]
A_27_P354322(r&s)	-2.7187808	Abscisic acid 8'-hydroxylase---Rep: Abscisic acid 8'-hydroxylase - Phaseolus vulgaris (Kidney bean) (French bean), partial (34%) [TC195807]
**C vs B down**		
A_27_P103166(r&s)	-3.7837956	Ethylene responsive transcription factor 2b---Rep: ERF-like protein - Cucumis melo (Muskmelon), partial (22%) [TC175128]
A_27_P056166(r&s)	-3.6210706	Ethylene-responsive transcription factor 1A---EST484069 GVN Medicago truncatula cDNA clone pGVN-69I8 5' end, mRNA sequence [BG582327]
A_27_P064221(r&s)	-2.2064414	PREDICTED: ethylene-responsive transcription factor ERF054-like---NF008B02ST1F1000 Developing stem Medicago truncatula cDNA clone NF008B02ST 5', mRNA sequence [AW688479]
A_27_P354322(r&s)	-1.7473531	Rep: Abscisic acid 8'-hydroxylase - Phaseolus vulgaris (Kidney bean) (French bean), partial (34%) [TC195807]
**D vs A down**		
A_27_P273002(r&l)	-3.7003493	Ethylene-regulated transcript 2 (ERT2) [Medicago truncatula]---EST611141 KVKC Medicago truncatula cDNA clone pKVKC-7D10, mRNA sequence [BQ165272]
A_27_P046491(r&s)	-2.4645548	Ethylene-responsive transcription factor ERF026---EST611182 KVKC Medicago truncatula cDNA clone pKVKC-7H4, mRNA sequence [BQ165313]
A_27_P216856(unknown)	-1.6135201	Rep: Cytokinin-specific binding protein - Vigna radiata, partial (77%) [TC184754]

A_27_P055546(r&l)	-2.569291	Cytokinin oxidase---EST333684 KV3 Medicago truncatula cDNA clone pKV3-22L6, mRNA sequence [AW774533]
A_27_P079281(r&s&l)	-2.5569634	Chitin-inducible gibberellin-responsive protein---MTYEA44TF JCVI-MT1 Medicago truncatula cDNA 5', mRNA sequence [EV260675]
A_27_P069601(r&s&l)	-2.250119	Chitin-inducible gibberellin-responsive protein---Rep: Chromosome undetermined scaffold_610, whole genome shotgun sequence - Vitis vinifera (Grape), partial (56%) [TC177482]
A_27_P066811(r&s&l)	-2.7124774	Chitin-inducible gibberellin-responsive protein---Rep: Chromosome undetermined scaffold_610, whole genome shotgun sequence - Vitis vinifera (Grape), partial (56%) [TC177482]
A_27_P354322(r&s)	-2.3516932	Abscisic acid 8'-hydroxylase---Rep: Abscisic acid 8'-hydroxylase - Phaseolus vulgaris (Kidney bean) (French bean), partial (34%) [TC195807]
**C vs A down**		
A_27_P273002(r&l)	-2.9610493	Ethylene-regulated transcript 2 (ERT2)---EST611141 KVKC Medicago truncatula cDNA clone pKVKC-7D10, mRNA sequence [BQ165272]
A_27_P055546(r&l)	-2.6926222	Cytokinin oxidase---EST333684 KV3 Medicago truncatula cDNA clone pKV3-22L6, mRNA sequence [AW774533]
A_27_P244732(unknown)	-1.5500085	Rep: Gibberellin oxidase-like protein - Arabidopsis thaliana (Mouse-ear cress), partial (38%) [TC196087]
A_27_P354322(r&s)	-1.5114269	Rep: Abscisic acid 8'-hydroxylase - Phaseolus vulgaris (Kidney bean) (French bean), partial (34%) [TC195807]

Table S3

Genes up- or down-regulated following exposure to acid-Al related to stress defense (FC ≥ 2.0; P < 0.05, *t* test).

A, B, C, and D represent the samples which were collected after germination treated with 0 (pH6.0), 0 (pH4.5), 0.8 (pH4.5), and 3.2 (pH4.5) mM $AlCl_3$ solution 60h, spectively.

Gene	Flod Chang	Description
**C vs A up**		
A_27_P128866(unknown)	2.4529815	Dehydration responsive element binding protein---Rep: DREB - Glycine max (Soybean), partial (44%) [TC200752]
A_27_P223101(sr)	2.2046506	Disease resistance response protein---Rep: Chromosome chr6 scaffold_3, whole genome shotgun sequence - Vitis vinifera (Grape), partial (70%) [TC199298]
A_27_P204676(sr)	2.0104442	Protein ALUMINUM SENSITIVE---Rep: Chromosome chr8 scaffold_29, whole genome shotgun sequence - Vitis vinifera (Grape), partial (98%) [TC200932]
A_27_P124741(unknown)	1.6329336	Rep: 22.0 kDa class IV heat shock protein precursor - Glycine max (Soybean), partial (90%) [TC185570]
A_27_P116426(unknown)	1.5184776	Rep: Disease resistance protein-like protein MsR1 - Medicago sativa (Alfalfa), partial (25%) [TC183856]
A_27_P130526(unknown)	2.0894868	Rep: Disease resistance response protein - Brassica campestris (Field mustard), partial (71%) [TC185405]
A_27_P348882(unknown)	1.886319	Rep: Universal stress protein - Medicago truncatula (Barrel medic), complete [TC190133]
A_27_P046846(sr)	2.722419	weakly similar to XP_003609593.1 Tir-nbs-lrr resistance protein, partial [Medicago truncatula]---EST636879 MTUS Medicago truncatula cDNA clone MTUS-8F12, mRNA sequence [CA919161]
**D vs A up**		
A_27_P128866(unknown)	7.475734	Dehydration responsive element binding protein---Rep: DREB - Glycine max (Soybean), partial (44%) [TC200752]
A_27_P046846(sr)	5.9765296	weakly similar to XP_003609593.1 Tir-nbs-lrr resistance protein, partial [Medicago truncatula]---EST636879 MTUS Medicago truncatula cDNA clone MTUS-8F12, mRNA sequence [CA919161]
A_27_P348882(unknown)	3.2568362	Universal stress protein A-like protein---Rep: Universal stress protein - Medicago truncatula (Barrel medic), complete [TC190133]

A_27_P204676(sr)	2.1342149	Protein ALUMINUM SENSITIVE---Rep: Chromosome chr8 scaffold_29, whole genome shotgun sequence - Vitis vinifera (Grape), partial (98%) [TC200932]
A_27_P130526(unknown)	2.0474153	Rep: Disease resistance response protein - Brassica campestris (Field mustard), partial (71%) [TC185405]

D vs B up

A_27_P204676(sr)	2.513399	Protein ALUMINUM SENSITIVE---Rep: Chromosome chr8 scaffold_29, whole genome shotgun sequence - Vitis vinifera (Grape), partial (98%) [TC200932]
A_27_P006131(r&l)	1.8808447	Rep: Heat shock protein DnaJ - Medicago truncatula (Barrel medic), complete [TC184311]
A_27_P039701(r&l)	3.061962	Resistance protein---EST611183 KVKC Medicago truncatula cDNA clone pKVKC-7H5, mRNA sequence [BQ165314]
A_27_P360357(unknown)	3.0684571	Rep: Functional candidate resistance protein KR1 - Glycine max (Soybean), partial (3%) [TC199377]

C vs B up

A_27_P007166(r&s&l)	2.625439	Disease resistance-like protein---AJ846367 MtSCF Medicago truncatula cDNA clone MtCF01P20S6, mRNA sequence [AJ846367]
A_27_P204676(sr)	2.3676379	Protein ALUMINUM SENSITIVE---Rep: Chromosome chr8 scaffold_29, whole genome shotgun sequence - Vitis vinifera (Grape), partial (98%) [TC200932]
A_27_P039701(r&s&l)	3.4249277	Resistance protein ---EST611183 KVKC Medicago truncatula cDNA clone pKVKC-7H5, mRNA sequence [BQ165314]
A_27_P360357(unknown)	1.7481853	Rep: Functional candidate resistance protein KR1 - Glycine max (Soybean), partial (3%) [TC199377]

D vs B down

A_27_P071426(ss)	-2.380004	TIR-NBS-LRR type disease resistance protein [Medicago truncatula]---NF056A08DT1F1065 Drought Medicago truncatula cDNA clone NF056A08DT 5', mRNA sequence [BQ144249]
A_27_P307167(r&s)	-5.6633787	18.2 kDa class I heat shock protein---EST510473 HOGA Medicago truncatula cDNA clone pHOGA-24E17 5' end, mRNA sequence [BG648844]
A_27_P101016(sr)	-6.9274735	Disease resistance response protein---Rep: Disease resistance-responsive family protein - Arachis hypogaea (Peanut), partial (59%) [TC175301]

A_27_P057546(other)	-2.1584842	Late embryongenesis abundant protein---EST635712 GLSD Medicago truncatula cDNA clone pGLSD-38E7, mRNA sequence [CA858457]
A_27_P124741(unknown)	-12.278862	Rep: 22.0 kDa class IV heat shock protein precursor - Glycine max (Soybean), partial (90%) [TC185570]
A_27_P052431(r&s&l)	-1.5673611	Rep: Heat shock factor protein HSF24 - Solanum peruvianum (Peruvian tomato) (Lycopersicon peruvianum), partial (23%) [TC196833]
A_27_P111726(r&s)	-1.7433794	Rep: Heat shock transcription factor - Medicago sativa (Alfalfa), partial (98%) [TC173663]
A_27_P207124(r&s&l)	-1.7134824	Rep: Heat-shock protein 80 - Euphorbia esula (Leafy spurge), partial (72%) [TC201521]
A_27_P091936(sr)	-2.993439	Resistance protein PLTR---EST507076 KV3 Medicago truncatula cDNA clone pKV3-46E22 5' end, mRNA sequence [BG645457]
A_27_P055582(r&s)	-2.7820044	Resistance protein---NF020A09EC1F1068 Elicited cell culture Medicago truncatula cDNA clone NF020A09EC 5', mRNA sequence [BF644737]
A_27_P036911(r&l)	-2.3064373	NBS-LRR resistance protein [Medicago truncatula]---NF010A11LF1F1084 Developing leaf Medicago truncatula cDNA clone NF010A11LF 5', mRNA sequence [AW683285]
A_27_P348882(unknown)	-10.950986	Universal stress protein A-like protein---Rep: Universal stress protein - Medicago truncatula (Barrel medic), complete [TC190133]
A_27_P132921(unknown)	-1.8099825	Rep: Desiccation protective protein LEA5 - Glycine max (Soybean), partial (53%) [TC186221]

C vs B down

A_27_P123906(sr)	-2.2176085	dehydration-responsive element-binding protein 1D---EST510552 HOGA Medicago truncatula cDNA clone pHOGA-24E16 5' end, mRNA sequence [BG648933]
A_27_P057546(other)	-2.8033476	Late embryongenesis abundant protein---EST635712 GLSD Medicago truncatula cDNA clone pGLSD-38E7, mRNA sequence [CA858457]
A_27_P124741(unknown)	-10.8172865	Rep: 22.0 kDa class IV heat shock protein precursor - Glycine max (Soybean), partial (90%) [TC185570]
A_27_P132921(unknown)	-1.9220167	Rep: Desiccation protective protein LEA5 - Glycine max (Soybean), partial (53%) [TC186221]
A_27_P101016(sr)	-1.9101572	Rep: Disease resistance-responsive family protein - Arachis hypogaea (Peanut), partial (59%) [TC175301]
A_27_P128866(unknown)	-2.2281926	Rep: DREB - Glycine max (Soybean), partial (44%) [TC200752]
A_27_P309937(sr)	-1.5447235	Rep: Embryonic abundant protein LEA-like - Arabidopsis thaliana (Mouse-ear cress), partial (5%) [TC189003]

A_27_P052431(r&s&l)	-1.5415554	Rep: Heat shock factor protein HSF24 - Solanum peruvianum (Peruvian tomato) (Lycopersicon peruvianum), partial (23%) [TC196833]
A_27_P326757(unknown)	-1.983692	Rep: NBS-LRR type disease resistance protein - Populus trichocarpa (Western balsam poplar) (Populus balsamiferasubsp. trichocarpa), partial (8%) [TC194100]
A_27_P348882(unknown)	-18.907494	Rep: Universal stress protein - Medicago truncatula (Barrel medic), complete [TC190133]
**D vs A down**		
A_27_P123906(sr)	-3.1218195	dehydration-responsive element-binding protein 1D [Zea mays]---EST510552 HOGA Medicago truncatula cDNA clone pHOGA-24E16 5' end, mRNA sequence [BG648933]
A_27_P101016(sr)	-2.534139	Disease resistance response protein---Rep: Disease resistance-responsive family protein - Arachis hypogaea (Peanut), partial (59%) [TC175301]
A_27_P257797(sr)	-2.1744177	Disease resistance-like protein GS4B-5, partial ---EST642315 GPOD Medicago truncatula cDNA clone GPOD-38O22, mRNA sequence [CA918168]
**C vs A down**		
A_27_P057546(other)	-2.1554835	Late embryongenesis abundant protein---EST635712 GLSD Medicago truncatula cDNA clone pGLSD-38E7, mRNA sequence [CA858457]
A_27_P037961(sr)	-3.4340773	TMV resistance protein N---EST509020 HOGA Medicago truncatula cDNA clone pHOGA-16F2 5' end, mRNA sequence [BG647401]

Table S4

Genes up- or down-regulated following exposure to acid-Al^{3+} are membrane transporters (FC ≥ 2.0; P < 0.05, *t* test).

A, B, C, and D represent the samples which were collected after germination treated with 0 (pH6.0), 0 (pH4.5), 0.8 (pH4.5), and 3.2 (pH4.5) mM $AlCl_3$ solution 60h, spectively.

Gene	Flod Chang	description
**C vs A up**		
A_27_P076296(unknown)	2.293866	ABC transporter B family member---AJ500424 MTGIM Medicago truncatula cDNA clone mtgmacc120016c04, mRNA sequence [AJ500424]
A_27_P116751(r&l)	2.0826585	ABC transporter I family member---Medicago truncatula clone MTYF9_FA_FB_FC1G-P-1 unknown mRNA [BT052075]
A_27_P253532(sr)	2.728354	Rep: Sulfate transporter protein-like - Oryza sativa subsp. japonica (Rice), partial (28%) [TC194607]
**D vs A up**		
A_27_P058051(unknown)	3.7986224	ABC transporter B family member [Medicago truncatula]---AJ499848 MTGIM Medicago truncatula cDNA clone mtgmacc120009c05, mRNA sequence [AJ499848]
A_27_P076296(unknown)	2.7189834	ABC transporter B family member---AJ500424 MTGIM Medicago truncatula cDNA clone mtgmacc120016c04, mRNA sequence [AJ500424]
A_27_P006741(sr)	4.791499	High-affinity nitrate transporter---EST649235 KV3 Medicago truncatula cDNA clone KV3-53P13, mRNA sequence [CB892266]
A_27_P099506(sr)	4.438461	High-affinity nitrate transporter---EST649235 KV3 Medicago truncatula cDNA clone KV3-53P13, mRNA sequence [CB892266]
A_27_P253532(sr)	4.454367	Rep: Sulfate transporter protein-like - Oryza sativa subsp. japonica (Rice), partial (28%) [TC194607]
**D vs B up**		
A_27_P058051(unknown)	2.0807507	ABC transporter B family member [Medicago truncatula]---AJ499848 MTGIM Medicago truncatula cDNA clone mtgmacc120009c05, mRNA sequence [AJ499848]
A_27_P047081(r&l)	2.33887	ABC transporter I family member---Medicago truncatula clone MTYF9_FA_FB_FC1G-P-1 unknown mRNA [BT052075]
A_27_P116751(r&l)	2.0588343	ABC transporter I family member---Medicago truncatula clone MTYF9_FA_FB_FC1G-P-1 unknown mRNA [BT052075]
A_27_P099506(sr)	13.509209	High-affinity nitrate transporter---EST649235 KV3 Medicago truncatula cDNA clone KV3-53P13, mRNA sequence

		[CB892266]
A_27_P006741(sr)	12.0869665	High-affinity nitrate transporter---EST649235 KV3 Medicago truncatula cDNA clone KV3-53P13, mRNA sequence [CB892266]
A_27_P284268(sr)	2.0869594	Peptide transporter PTR1---Rep: Chromosome undetermined scaffold_53, whole genome shotgun sequence - Vitis vinifera (Grape), partial (42%) [TC197247]
A_27_P204126(sr)	2.1722283	Zinc transporter---Medicago truncatula metal transport protein (ZIP6) mRNA, complete cds [AY339058]
A_27_P127831(unknown)	1.658685	Rep: ABC transporter related; Choline dehydrogenase - Medicago truncatula (Barrel medic), complete [TC183772]
**C va B up**		
A_27_P116751(r&l)	2.4245462	ABC transporter I family member---Medicago truncatula clone MTYF9_FA_FB_FC1G-P-1 unknown mRNA [BT052075]
A_27_P047081(r&l)	2.3403769	ABC transporter I family member---Medicago truncatula clone MTYF9_FA_FB_FC1G-P-1 unknown mRNA [BT052075]
A_27_P099506(sr)	3.8165526	High-affinity nitrate transporter ---EST649235 KV3 Medicago truncatula cDNA clone KV3-53P13, mRNA sequence [CB892266]
A_27_P006741(sr)	4.2978263	High-affinity nitrate transporter---EST649235 KV3 Medicago truncatula cDNA clone KV3-53P13, mRNA sequence [CB892266]
A_27_P055076(sr)	2.3163261	Nitrate transporter---EST392314 DSIL Medicago truncatula cDNA clone pDSIL-12C15, mRNA sequence [AW981224]
A_27_P068851(s&l)	3.5400069	Nitrate transporter---EST530430 GPOD Medicago truncatula cDNA clone pGPOD-10M12 5' end, mRNA sequence [BI309020]
A_27_P305257(sr)	2.246306	Nitrate transporter---Rep: Chromosome chr2 scaffold_241, whole genome shotgun sequence - Vitis vinifera (Grape), partial (33%) [TC199130]
A_27_P104651(sr)	2.1013725	Peptide transporter PTR2---Rep: Chromosome chr8 scaffold_29, whole genome shotgun sequence - Vitis vinifera (Grape), partial (91%) [TC186239]
A_27_P204126(sr)	2.1180222	Zinc transporter---Medicago truncatula metal transport protein (ZIP6) mRNA, complete cds [AY339058]
A_27_P315097(sl)	1.5194438	Rep: High affinity potassium transporter 2 - Mesembryanthemum crystallinum (Common ice plant), partial (28%) [TC191134]
A_27_P311187(unknown)	1.9941057	ABC transporter---Rep: Chromosome chr10 scaffold_43, whole genome shotgun sequence - Vitis vinifera (Grape), partial (39%) [TC202960]

**C vs B down**		
A_27_P133281(sr)	-8.404731	ABC transporter B family member---Rep: Chromosome chr5 scaffold_2, whole genome shotgun sequence - Vitis vinifera (Grape), partial (42%) [TC178161]
A_27_P342407(sr)	-20.217505	ABC transporter B family member---Rep: Chromosome chr7 scaffold_42, whole genome shotgun sequence - Vitis vinifera (Grape), partial (32%) [TC180761]
A_27_P024026(unknown)	-10.3139	Rep: Mitochondrial phosphate transporter - Glycine max (Soybean), partial (81%) [TC184453]
**D vs B down**		
A_27_P133281(sr)	-10.068896	ABC transporter B family member---Rep: Chromosome chr5 scaffold_2, whole genome shotgun sequence - Vitis vinifera (Grape), partial (42%) [TC178161]
A_27_P342407(sr)	-27.504162	ABC transporter B family member---Rep: Chromosome chr7 scaffold_42, whole genome shotgun sequence - Vitis vinifera (Grape), partial (32%) [TC180761]
A_27_P024026(unknown)	-8.96609	Rep: Mitochondrial phosphate transporter - Glycine max (Soybean), partial (81%) [TC184453]
**C vs A down**		
A_27_P128161(r&s&l)	-2.1637785	Nitrate transporter NTL1---Rep: Nitrate transporter (NTL1); 53025-56402 - Arabidopsis thaliana (Mouse-ear cress), partial (60%) [TC193814]
**D vs A down**		
A_27_P128161(r&s&l)	-1.6182357	Rep: Nitrate transporter (NTL1); 53025-56402 - Arabidopsis thaliana (Mouse-ear cress), partial (60%) [TC193814]
A_27_P315097(sl)	-1.919894	Rep: High affinity potassium transporter 2 - Mesembryanthemum crystallinum (Common ice plant), partial (28%) [TC191134]

Table S5

Detail information of high significant enrichment GO terms list selected with p-value < 1×10^{-3}, FDR < 0.05.

GO_acc	Ontology	Description	Number in input list	Number in BG/Ref	p-value	FDR
GO:0000096	P	response to carbohydrate stimulus	34	174	1.60E-28	2.00E-25
GO:0002252	P	immune effector process	8	33	9.40E-09	3.00E-07
GO:0002376	P	immune system process	22	354	5.10E-09	1.70E-07
GO:0003677	F	DNA binding	46	1389	9.20E-08	2.50E-06
GO:0003700	F	transcription factor activity	46	944	7.20E-13	1.80E-10
GO:0004553	F	hydrolase activity, hydrolyzing O-glycosyl compounds	21	311	2.60E-09	1.60E-07
GO:0004601	F	peroxidase activity	12	98	8.40E-09	3.00E-07
GO:0005506	F	iron ion binding	9	118	2.90E-05	0.00066
GO:0005516	F	calmodulin binding	9	160	0.0003	0.0046
GO:0005576	C	extracellular region	10	184	0.00019	0.028
GO:0006139	P	nucleobase, nucleoside, nucleotide and nucleic acid metabolic process	46	1727	2.20E-05	0.00031
GO:0006350	P	transcription	41	927	1.70E-10	6.50E-09
GO:0006351	P	transcription, DNA-dependent	23	484	3.00E-07	6.80E-06
GO:0006355	P	regulation of transcription, DNA-dependent	23	445	6.90E-08	1.80E-06
GO:0006519	P	cellular amino acid and derivative metabolic process	46	542	9.30E-22	4.00E-19
GO:0006555	P	methionine metabolic process	6	39	1.10E-05	0.00019
GO:0006575	P	cellular amino acid derivative metabolic process	33	276	8.40E-21	2.20E-18
GO:0006629	P	lipid metabolic process	18	593	0.0012	0.012
GO:0006952	P	defense response	40	703	1.50E-13	1.10E-11

GO:0006955	P	immune response	22	351	4.40E-09	1.50E-07
GO:0008610	P	lipid biosynthetic process	11	311	0.0031	0.028
GO:0009055	F	electron carrier activity	11	195	6.70E-05	0.0012
GO:0009058	P	biosynthetic process	89	2714	2.60E-12	1.40E-10
GO:0009059	P	macromolecule biosynthetic process	46	1694	1.40E-05	0.00022
GO:0009536	C	plastid	25	91	1.50E-04	0.017
GO:0009611	P	response to wounding	10	188	0.00022	0.0027
GO:0009698	P	phenylpropanoid metabolic process	27	185	1.10E-19	2.10E-17
GO:0009699	P	phenylpropanoid biosynthetic process	25	160	4.20E-19	6.80E-17
GO:0009743	P	response to carbohydrate stimulus	34	174	1.60E-28	2.00E-25
GO:0009812	P	flavonoid metabolic process	18	116	3.20E-14	2.30E-12
GO:0009813	P	flavonoid biosynthetic process	18	107	7.50E-15	6.10E-13
GO:0009889	P	regulation of biosynthetic process	46	921	3.10E-13	1.80E-11
GO:0009891	P	positive regulation of biosynthetic process	7	62	1.80E-05	0.00026
GO:0009893	P	positive regulation of metabolic process	7	71	4.30E-05	0.00059
GO:0010033	P	response to organic substance	52	896	3.70E-17	3.70E-15
GO:0010200	P	response to chitin	27	99	2.60E-27	1.70E-24
GO:0010467	P	gene expression	45	1620	9.80E-06	0.00016
GO:0010468	P	regulation of gene expression	41	951	3.50E-10	1.30E-08
GO:0010556	P	regulation of macromolecule biosynthetic process	41	873	2.90E-11	1.30E-09
GO:0010557	P	positive regulation of macromolecule biosynthetic process	5	54	0.00071	0.0076
GO:0010604	P	positive regulation of macromolecule metabolic process	5	55	0.00077	0.0081

GO:0010628	P	positive regulation of gene expression	5	51	0.00055	0.0061
GO:0016209	F	antioxidant activity	13	118	7.60E-09	3.00E-07
GO:0016563	F	transcription activator activity	9	129	5.90E-05	0.0011
GO:0016684	F	oxidoreductase activity, acting on peroxide as acceptor	12	98	8.40E-09	3.00E-07
GO:0016798	F	hydrolase activity, acting on glycosyl bonds	23	343	5.70E-10	4.70E-08
GO:0019219	P	regulation of nucleobase, nucleoside, nucleotide and nucleic acid metabolic process	41	876	3.20E-11	1.40E-09
GO:0019222	P	regulation of metabolic process	47	1109	4.30E-11	1.80E-09
GO:0019438	P	aromatic compound biosynthetic process	27	227	2.30E-17	2.70E-15
GO:0019748	P	secondary metabolic process	34	387	3.30E-17	3.60E-15
GO:0020037	F	heme binding	8	79	1.00E-05	0.00025
GO:0030528	F	transcription regulator activity	50	1122	2.20E-12	2.70E-10
GO:0031323	P	regulation of cellular metabolic process	47	1019	2.70E-12	1.40E-10
GO:0031325	P	positive regulation of cellular metabolic process	7	71	4.30E-05	0.00059
GO:0031326	P	regulation of cellular biosynthetic process	46	921	3.10E-13	1.80E-11
GO:0031328	P	positive regulation of cellular biosynthetic process	7	62	1.80E-05	0.00026
GO:0032774	P	RNA biosynthetic process	23	485	3.10E-07	6.90E-06
GO:0034645	P	cellular macromolecule biosynthetic process	46	1663	9.00E-06	0.00015

GO:0042221	P	response to chemical stimulus	73	1477	5.80E-19	8.30E-17
GO:0042398	P	cellular amino acid derivative biosynthetic process	30	218	7.10E-21	2.20E-18
GO:0044249	P	cellular biosynthetic process	89	2583	2.20E-13	1.50E-11
GO:0044255	P	cellular lipid metabolic process	14	407	0.0013	0.012
GO:0044283	P	small molecule biosynthetic process	40	575	2.40E-16	2.10E-14
GO:0045087	P	innate immune response	19	333	1.90E-07	4.70E-06
GO:0045449	P	regulation of transcription	41	842	1.00E-11	4.80E-10
GO:0045935	P	positive regulation of nucleobase, nucleoside, nucleotide and nucleic acid metabolic process	5	54	0.00071	0.0076
GO:0045941	P	positive regulation of transcription	5	51	0.00055	0.0061
GO:0046906	F	tetrapyrrole binding	8	105	8.00E-05	0.0013
GO:0048518	P	positive regulation of biological process	11	201	8.80E-05	0.0012
GO:0048522	P	positive regulation of cellular process	8	131	0.00037	0.0043
GO:0050789	P	regulation of biological process	67	2043	4.90E-10	1.80E-08
GO:0050794	P	regulation of cellular process	63	1815	1.70E-10	6.50E-09
GO:0050896	P	response to stimulus	119	3029	2.70E-20	5.80E-18
GO:0051171	P	regulation of nitrogen compound metabolic process	42	902	2.30E-11	1.10E-09
GO:0051173	P	positive regulation of nitrogen compound metabolic process	5	54	0.00071	0.0076
GO:0051252	P	regulation of RNA metabolic process	23	448	7.80E-08	2.00E-06
GO:0060255	P	regulation of macromolecule metabolic process	41	985	9.40E-10	3.30E-08
GO:0065007	P	biological regulation	76	2381	1.70E-10	6.50E-09

GO:0080090	P	regulation of primary metabolic process	46	956	1.10E-12	6.20E-11

Table S6

List of the metabolic pathways of 226 genes which belonged to significant enrichment GO terms.

Pathways	Sequences	enzymes
Phenylpropanoid biosynthesis	17	6
Phenylalanine metabolism	14	3
Starch and sucrose metabolism	13	7
Galactose metabolism	6	4
Other glycan degradation	6	3
Purine metabolism	6	2
Glycosaminoglycan degradation	5	2
Thiamine metabolism	5	1
Steroid hormone biosynthesis	5	2
Glycosphingolipid biosynthesis-ganglio series	4	1
Porphyrin and chlorophyll metabolism	4	3
Drug metabolism-cytochrome P450	4	2
Sphingolipid metabolism	4	1
Glutathione metabolism	4	3
Metabolism of xenobiotics by cytochrome P450	4	2
Cysteine and methionine metabolism	4	5
Flavonoid biosynthesis	4	3
Tryptophan metabolism	4	3
Steroid degradation	3	1
Cyanoamino acid metabolism	3	3
Pentose and glucuronate interconversions	3	1
Aminoacyl-tRNA biosynthesis	3	3
Linoleic acid metabolism	3	2
Arachidonic acid metabolism	3	2
Selenocompound metabolism	3	4

Aminobenzoate degradation	3	2
Fatty acid degradation	3	2
One carbon pool by folate	2	1
Glycerolipid metabolism	2	2
Caffeine metabolism	2	1
Sulfur metabolism	2	2
alpha-Linolenic acid metabolism	2	2
Retinol metabolism	2	1
Taurine and hypotaurine metabolism	1	1
Caprolactam degradation	1	1
Benzoate degradation	1	1
beta-Alanine metabolism	1	2
Nitrogen metabolism	1	1
Butanoate metabolism	1	1
N-Glycan biosynthesis	1	1
Stilbenoid, diarylheptanoid and gingerol biosynthesis	1	2
Flavone and flavonol biosynthesis	1	1
Ubiquinone and other terpenoid-quinone biosynthesis	1	1
Isoflavonoid biosynthesis	1	1
Glyoxylate and dicarboxylate metabolism	1	1
Anthocyanin biosynthesis	1	1
Fatty acid elongation	1	1
Inositol phosphate metabolism	1	1
Zeatin biosynthesis	1	1
Carotenoid biosynthesis	1	1
Tyrosine metabolism	1	1

Diterpenoid biosynthesis	1	2
Limonene and pinene degradation	1	1
Geraniol degradation	1	1
Valine, leucine and isoleucine degradation	1	2
Streptomycin biosynthesis	1	1
Styrene degradation	1	1
Amino sugar and nucleotide sugar metabolism	1	2
Propanoate metabolism	1	2
Cutin, suberine and wax biosynthesis	1	1
Isoquinoline alkaloid biosynthesis	1	1
Glycine, serine and threonine metabolism	1	1
Biosynthesis of unsaturated fatty acids	1	1
Lysine degradation	1	1
